# Implementing a revised online screening tool in a routine care online clinic treating anxiety and depression

**DOI:** 10.3389/fdgth.2023.1128893

**Published:** 2023-07-12

**Authors:** Kim Mathiasen, Trine Theresa Holmberg Sainte-Marie, Helene Skaarnes, Esben Kjems Jensen, Christiaan Vis, Kristine Tarp

**Affiliations:** ^1^ Research Unit for Digital Psychiatry, Centre for Digital Psychiatry, Mental Health Services of Southern Denmark, Odense, Denmark; ^2^ Department of Clinical Research, Faculty of Health Sciences, University of Southern Denmark, Odense C, Denmark; ^3^ Clinical, Neuro-, & Developmental Psychology, Faculty of Behavioural and Movement Sciences, VU Amsterdam, Amsterdam, Netherlands; ^4^ Amsterdam Public Health Research Institute, Amsterdam, Netherlands; ^5^ Section for Research-Based Innovation, Division of Psychiatry, Haukeland University Hospital, Bergen, Norway

**Keywords:** implementation, internet-based cognitive behavioral therapy, screening optimization, anxiety, depression

## Abstract

**Introduction:**

The ItFits implementation toolkit was developed as part of the ImpleMentAll EU Project, to help guide implementation processes. The ItFits toolkit was tested in the online clinic, Internetpsykiatrien, in the Region of Southern Denmark, where it was employed to optimize screening and intake procedures. We hypothesized that a larger proportion of assessed patients would be referred to treatment. Further, we hypothesized the completion rate and effectiveness would increase, as a result of including a more relevant sample.

**Method:**

Using the ItFits-toolkit, Internetpsykiatrien developed a revised online screening tool. Data on patient flow and symptom questionnaires was extracted from Internetpsykiatrien six months prior to- and six months after implementation of the revised online screening tool.

**Results:**

A total of 1,830 applicants self-referred for treatment during the study period. A significantly lower proportion of patients were referred to treatment after implementation of the revised screening tool (pre-implementation, *n* = 1,009; post-implementation, *n* = 821; odds ratio 0.67, 95% CI: 0.51; 0.87). The number of patients that completed treatment increased significantly (pre-implementation: 136/275 [49.45%], post-implementation, *n* = 102/162 [62.96%]; odds ratio 1.79, 95% CI 1.20; 2.70). The treatment effect was unchanged (*B* = 0.01, *p *= .996). Worth noting, the number of patients that canceled their appointment for the video assessment interview decreased drastically.

**Conclusion:**

By using the ItFits toolkit for a focused and structured implementation effort, the clinic was able to improve the completion rate, which is an important effect in iCBT. However, contrary to our hypotheses, we did not find an increase in clinical effect, nor a larger ratio being referred to treatment after assessment. The decreased number of referrals for treatment could be a result of increased awareness of inclusion criteria among the clinicians.

## Introduction

### Prevalence and cost of anxiety and depression

Depression and anxiety disorders are both highly prevalent in Europe and Denmark with 14 percent of the population reporting a mood disorder (1, 2). In Europe affective disorders (depression and bipolar affective disorder) are estimated at 21 million cases whereas anxiety disorders amount to 41 million cases (3). The cost of affective disorders in the EU was 106 billion euros in 2004, whereas anxiety disorders cost the EU countries 41 billion euros (3).

Patients with mood disorders experience a significant loss of Quality Adjusted Life-Years (QALYs) compared with chronic disorders (4). Overall, 1,831 QALYs are lost per 100,000 patients {Grandes, 2011 #10}. Moreover, compared with the general population, patients with mental disorders experience one standard deviation lower mental quality of life (4). This emphasizes the need for easy and accessible evidence-based interventions to treat and support patients with mood disorders.

### Internet-based cognitive behavioral therapy

Among the barriers to help-seeking behavior are high costs of psychotherapy, stigma associated with mental illness, lack of trained clinicians, and limited access to low cost and evidence-based psychotherapy (5). Psychotherapy, such as cognitive behavioral therapy (CBT), is effective for treating anxiety and depression (6–8), but dissemination has proven to be an obstacle (9). Several researchers recommend further study of implementation and dissemination of evidence-based psychotherapies to alleviate the issues (9–11).

Over the past two decades, researchers have been investigating the possibility of delivering CBT via the Internet in a guided self-help format (iCBT) as a means of enhancing the reach of CBT. Several meta-analyses have shown this treatment format to be efficacious for depression and anxiety disorders (12–15).

The problem is that novel technology and health care services are hard to implement. On average new treatments take 17 years to become normalized in routine care service delivery (16). One of the barriers which can obstruct the dissemination of iCBT is the implementation process itself (17, 18).

### Tailored intervention in an iCBT clinic

There are several known barriers to the implementation of Internet-based treatments for mental disorders. These include, among others, lack of awareness of, and negative attitudes towards Internet-based treatment among service providers and patients, high drop-out rates from treatment, limited availability of trained professionals, and limited evidence for cost-effectiveness (19). Use of a modified delivery of iCBT thus requires an implementation process. That is, a process where interventions are integrated and made workable in praxis (20).

The purpose of implementation is normalization. Normalization refers to a new working procedure entering a state of normality in the minds of all individuals involved (21). When working with implementation processes in an iCBT clinic, tools to guide the local implementation team may assist in qualifying the implementation processes, and thus enhance the progression of the normalization process.

Up until now, implementation has mostly been expert-driven. Expert-driven implementation consists of experienced implementation researchers or implementation practitioners having a prominent role in guiding the process, as well as designing and applying the implementation strategy. Usually, these experts are external to the implementation site, and may therefore not be familiar with the specific context or the intervention that is to be implemented. This may lead to less effective implementation strategies (22). Therefore, it may be important to involve local implementers in tailoring the implementation process. This is referred to as self-guided tailoring, or tailored implementation.

Tailored implementation interventions are designed to achieve changes in healthcare practices, based on an assessment of a particular healthcare practice, and their desires for change (23). Innovations can be implemented quicker and more efficiently by using tailored implementation strategies that systematically address the factors that are most likely to obstruct or facilitate normalization in a local setting (23, 24).

The Integrated Theory-based Framework for Intervention Tailoring Strategies toolkit (ItFits-toolkit) was developed to help deliver tailored implementation interventions (21, 25). The toolkit is an online, self-guided tool consisting of four modules that guides the users through the tailoring process of the implementation. The ItFits-toolkit is developed for local implementers and provides a systematic and flexible approach rooted in theoretical and conceptual ideas from the field of implementation science and has shown promising results on normalization (25).

## Aims

The aim of the present study was to investigate the effects of a specific use case of the ItFits toolkit, i.e., to determine, whether the toolkit was effective as a tool based on the local goal set by a local team of implementers in the iCBT clinic Internetpsykiatrien. The most resource heavy single part of the service is to provide video-based assessment interviews, so the clinic wished to develop an improved online screening prior to the assessment as a part of the self-referral process. The aim was to include a more relevant sample to the assessment interviews and by extension to the treatment. In other words, they worked with an implementation effort on patient intake in the routine care iCBT clinic Internetpsykiatrien, following an implementation intervention guided by the ItFits-toolkit.

In the present study, the following three hypotheses were tested:

1) First, we hypothesized that larger proportion of assessed patients would be referred to iCBT treatment.
  The rationale behind this is that less selection would take place during the clinical interview, as some patients who would have been previously gone through the screening, would themselves chose other treatment, or would be referred elsewhere during screening.
2) Our second hypothesis was that the completion rate would be increased.
  This was hypothesized based on that the demographic going through treatment, would benefit from and be suited for the program.
3) Finally, we hypothesized that the clinical effectiveness would be increased after implementation.
  We proposed this hypothesis based on the assumption that in the “before” setting, part of the sample may have been less suited for this specific treatment style and method, meaning that even if they completed it, they may have benefitted less. The theoretical foundation for this is that therapy may be less efficacious in cases of misdiagnosis, or if patients are not compliant ({Kingdon, 2007 #38}{Howes, 2022 #36}), wherefore sharpening the inclusion process may minimize the risk of including persons who will not benefit from treatment.

## Methods and materials

### Design

The study was designed as a pragmatic, retrospective cohort study, investigating real-world data from the routine care clinic Internetpsykiatrien situated in Odense, Denmark. The clinic provides national treatment for mild to moderate anxiety and depression, and treatment is free of charge for the users.

The new online screening tool for Internetpsykiatrien was implemented on November 8 2019. Data from 6 months prior to implementation (May 8 2019–November 7 2019) and 6 months after implementation (November 8 2019–May 8 2020) were extracted from the clinic to analyze the effects of the screening tool.

### Ethics

The study was part of the clinic's quality assessment efforts, therefore, ethical clearance by Danish standards is not applicable, since the present sub-study, was solely based on questionnaire data from the treatment study. The study is conducted in accordance with the ethical standards of the institutional and national research committee and with the 1964 Helsinki Declaration and its later amendments or comparable ethical standards. All patients gave digital written informed consent for their treatment data to be used for research purposes. The study was reported to the Danish Data Protection Agency.

**The ItFits-toolkit** consists of an online program, which guides an implementation team through four modules to work systematically with creating, executing and evaluating a tailored implementation effort. The four modules comprise: of (1) Identification and prioritization of implementation goals and determinants of practice. The latter refers to barriers and facilitators for the implementation process. (2) Matching implementation strategies to the determinants of practice. This comprises selecting one or more actions or strategies to improve implementation, which can either be selected from a set of in-built evidence-informed example strategies or the team can devise their own strategy. (3) A concrete plan is then designed for carrying out the strategies, (4) which in the final module is applied and evaluated. The process can then be repeated. The ItFits-toolkit was developed and tested in a large-scale European project (ImpleMentAll.eu) with promising results (Vis et al. in prep.).

**Patient Health Questionnaire-9** (PHQ-9) (26, 27). The PHQ-9 is a nine-item mood module, which can be used to screen for the presence of depressive symptoms. The nine items are each scored on a 0–3 scale with the total score ranging from 0 to 27 and higher scores indicating more severe depression. The PHQ-9 has been shown to have good psychometric properties (28).

**Generalized Anxiety Disorder Assessment** (GAD-7) (29, 30). The GAD-7 was used to measure symptoms of anxiety. It consists of seven items scored on a 0–3 scale with a total range of 0–21, with higher scores indicating higher severity levels. While the GAD-7 was originally developed to measure Generalized Anxiety Disorder, it has also been validated as a general measure of symptom severity of anxiety across different anxiety disorders in heterogeneous samples (31).

### Recruitment and inclusion criteria

Internetpsykiatrien operates as part of routine care in Denmark with national uptake and is funded by the five Danish health regions, which are tax-funded public authorities. The clinic uses self-referral via a website (Internetpsykiatrien.dk), where patients fill out a secure application form. They do not need referrals from other sources such as a general practitioner. Treatment, including the use of the programs, is free of charge for the patients. After an online screening of their application, those eligible are invited to a video-based assessment interview with a licensed psychologist or a psychologist under the supervision of a licensed psychologist. If parts of the application give rise to concerns, applicants are contacted for clarification by telephone and, if appropriate (e.g., in cases of increased suicidal risk), provided access to more relevant sources of assistance. Denmark is well suited for internet-based screening and treatment, since an estimated 97% of citizens have access to the internet, and 91% have a personal computer (32).

Eligibility criteria for the clinic are: age ≥18 years; meeting the diagnostic criteria of the International Classification of Diseases and Related Health Problems, 10th edition (33), for major depressive disorder, panic disorder, agoraphobia, social phobia, specific phobia; not currently at high risk of suicide; no comorbid substance dependence, bipolar affective disorder, psychotic illness, or obsessive-compulsive disorder; not currently undergoing other psychological treatment for depression or anxiety; access to a personal computer and fast internet connection; and adequate understanding of spoken and written Danish.

After the assessment, the patients are provided access to an online treatment program for their specific disorder. Additionally, they are provided weekly or biweekly clinical support from a licensed clinical psychologist or a psychologist under the supervision of a licensed psychologist. The cadence of support is based on the how fast the patient progresses through exercises. If a patient completes an exercise weekly, they get weekly feedback. If they are inactive for more than a week, the psychologist reaches out to them themselves. Support is provided via an in-built secure text module.

### Screening tools

Internetpsykiatrien participated as a study site in the ImpleMentAll project. The focal area chosen was to improve the initial screening in the self-referral procedure in order to include a more relevant population. This is of importance to the clinic since the assessment interviews are the single most resource-demanding task in the service. From an economic standpoint, the self-referrals should be as relevant as possible to preserve resources for the relevant target demographic.

The strategy chosen for the primary goal “that the patients who seek treatment are eligible” was to develop a more comprehensive and sophisticated online screening as part of the self-referral procedure.

#### The original screening tool

Prior to implementation, the online screening tool included the following elements:

A brief description of the self-referral process.

Background information for the applicant (social security number, name, address, contact information, civil status, number of children, level of education, job status).

Open ended questions with free-text response:

a) “What is the reason for your application? What do you want help with?”
b) “What made you choose Internetpsykiatrien instead of another treatment?”
c) “What do you expect of online treatment?”
d) “Do you have any additional comments?”

Duration of any known anxiety or depressive disorder.

Whether their general physician was aware to their problems, and if they had been formally diagnosed.

Use of:

a) Psychiatric medication.
b) Non-pharmacological psychiatric or psychological treatment.
c)  Alchol.
d) Drugs.

Questionnaires relating to depression and anxiety:

a) Patient health questionnaire-9 (PHQ-9) for depressive symptoms.
b) Fear Questionnaire (FQ) for anxiety symptoms.

#### The revised screening tool

The online screening tool was updated to include the following elements:

An improved text-based introduction with descriptions of:

a) What to expect from the assessment interview, and what the clinician expects of the applicant.
b) Which diagnoses and severity levels are covered by the clinic; depression, panic disorder (with and without agoraphobia), social phobia, or specific phobia of mild to moderate severity.
c) The service as a self-administered online treatment program including daily exercises and questionnaires about the condition including how data is stored.

Questionnaires relating to each of the four disorders treated at Internetpsykiatrien:

a) Patient Health Questionnaire-9 (PHQ-9) for depressive symptoms.
b) Panic Disorder Severity Scale-Self Report (PDDS-SR) for panic disorder with or without agoraphobia.
c) Social Interaction Anxiety Scale (SIAS) for social anxiety disorder
d) Fear Questionnaire (FQ) for specific phobias.

Open ended questions with free-text response:

a) “What made you choose internet-based treatment instead of another type of treatment?”
b) “What do you expect of internet-based treatment?”
c) “Describe in your own words, why you are applying for treatment at Internetpsykiatrien.”
d) “Do you have any additional comments?”

A series of questions probing various circumstances that would exclude the patient from treatment at Internetpsykiatrien. These circumstances result in automatic exclusion without requiring a screening by a psychologist:

a) Presence of the following diagnoses: bipolar disorder, schizophrenia, obsessive compulsive disorder personality disorder
b) Presence of untreated post-traumatic stress disorder.
c) Weekly alcohol consumption >20 units.
d) Not having access to computer or tablet with a webcam.
e) Currently receiving psychological treatment elsewhere.
f) Only interested in consultations with a psychologist and not internet-based guided treatment.
g) Applying for treatment for a disorder that is not anxiety or depression.

The revised screening tool was tested during the ImpleMentAll trial. No additional pilot study was conducted.”

### Statistical analyses

The sample was described using descriptive statistics, and the groups were compared using *t*-tests for continuous variables or Chi-square tests for categorical variables. Additionally, a flow chart was plotted.

The primary hypothesis was tested using logistic regression. The outcome variable was a binary variable of having initiated treatment in the program. Exposure was likewise defined in a binary variable of either belonging to the pre-implementation group or to the post-implementation group. Age, gender, income, highest level of education and having children were included as potential confounders in the model. Adjusted and non-adjusted results were reported as well as the number of patients in each group who initiated treatment and the proportion.

Similarly, we applied a logistic regression analysis to test for the secondary hypothesis. Completion was used as the outcome variable and group belonging as the exposure variable. We used the same confounder variables as above. Completion was defined following the clinic's definition: the patient must either have completed all modules in the program or have been active in the treatment for 12 consecutive weeks or both.

To test the third hypothesis, a linear regression model was used. As an outcome variable, the difference in symptomatic level from pre- to post-treatment on the outcome measure of the patients’ primary disorders was used (PHQ-9 or GAD-7). In addition to the confounding variables described above, the baseline severity level was included in the model. Adjusted and non-adjusted difference of differences were reported along with the mean change and standard deviations.

All analyses were performed using R version 4.1.1 and R-studio version 1.4.1717 (34) (REF: R Core group (2021). For description of sample, the package tableone was used (35).

## Results

### Description of the sample

**Figure 1:**
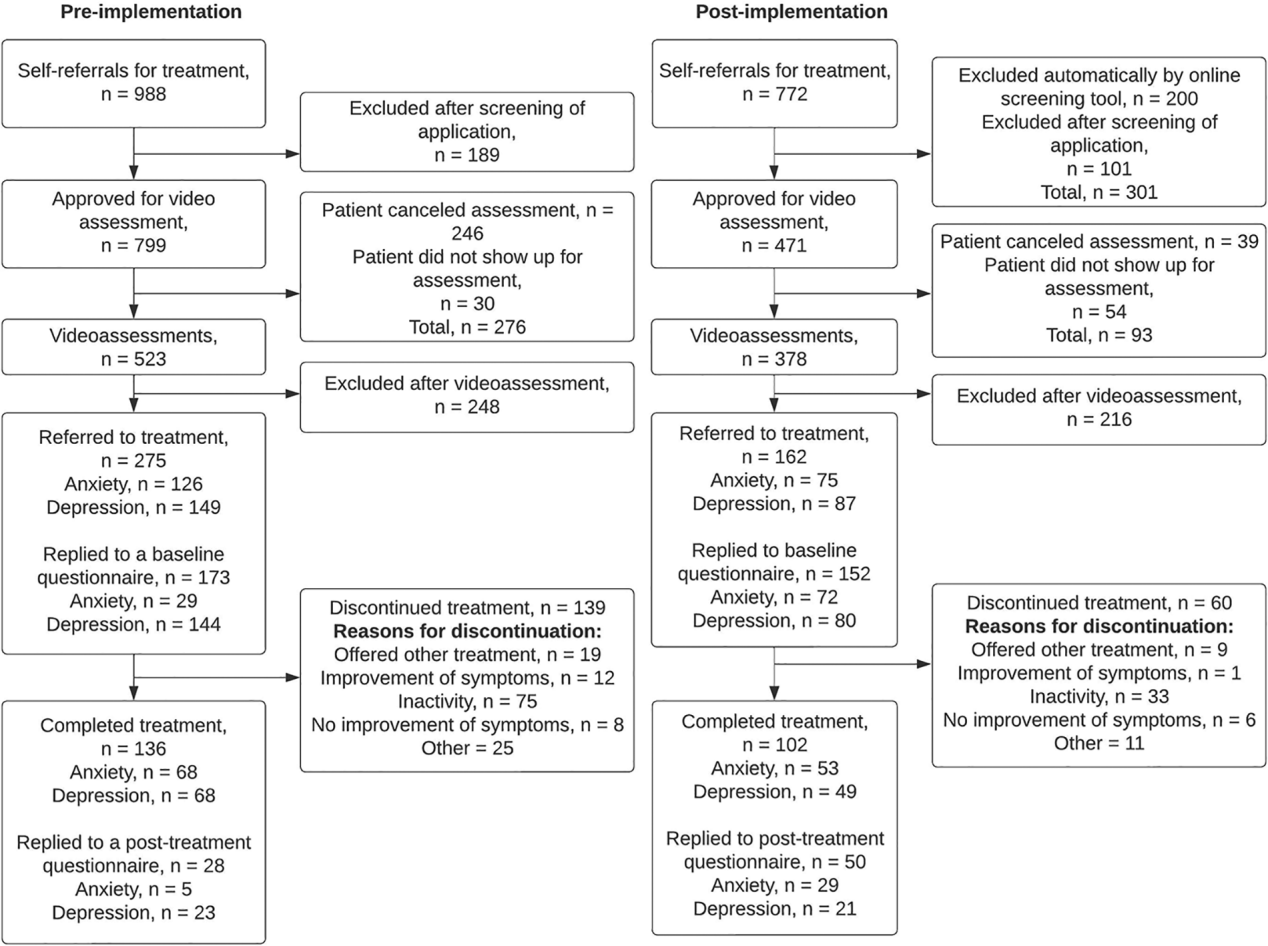
Patient flow before and after implementation of the new screening procedure.

Internetpsykiatrien had a total of *N* = 1830 self-referrals during the study period. Of these, *n* = 1009 applied for treatment prior to implementation of the revised online screening tool, and *n* = 821 applied for treatment after implementation of the online screening tool. The revised online screening tool automatically excluded *n* = 200 ineligible applicants. No data was stored on automatically excluded applications. Thus, they are not included in any further analyses (although they are included in the flowchart, Figure 1). Additionally, *n* = 70 applicants (*n* = 21 pre-implementation; *n* = 49 post-implementation), were excluded from analyses due to registration errors in the dataset, making it uninterpretable at which stage they were excluded from treatment (these applicants are not included in the flowchart). Thus, the final dataset for this study included *n* = 1,560 self-referrals (n = 988 pre-implementation; *n* = 572 post-implementation).

After the implementation of the new online screening tool, Internetpsykiatrien received a lower number of self-referrals than prior to implementation (772 vs. 988). After implementation, fewer of the self-referrals were approved for a video assessment (61.0% vs. 80.9%). However, a larger proportion of the approved video assessments were actualized (80.3% vs. 65.5%). Prior to implementation, *n* = 246 assessments (30.8%) were canceled by the patient, whereas after implementation, only 39 assessments (8.3%) were canceled by the patient.

Symptom questionnaires were not implemented at the same time for anxiety and depression treatment programs. Therefore, only a small proportion of anxiety patients received questionnaires prior to the implementation of the new screening tool. As a result, only *n* = 29 of *n* = 126 (23%) anxiety patients replied to the baseline questionnaire prior to implementation. Replies to post-treatment questionnaires were generally low, with *n* = 28 replies out of *n* = 136 (20.6%) completed treatments prior to implementation, and *n* = 50 replies out of *n* = 102 (49.0%) completed treatment after implementation.

Table 1 describes the two samples of all applicants to iCBT treatment in the clinic Internetpsykiatrien 6 months prior to or after implementation of the new screening procedure, respectively. The two samples were similar in mean age, gender, whether they had children, and in their marital status. However, they differed in highest level of education and in source of income. Regarding the highest level of education, fewer reported primary school as the highest level in the sample after the implementation of the new screening procedure. More reported high school as the highest level of education, which showed the biggest difference between samples (19.1% vs. 24.5%). Furthermore, the after-sample reported more intermediate further education and fewer higher. When regarding the source of income, a larger proportion of the after-sample reported being employed (33.7% vs. 40.9%) or receiving a stipendium (indicating they were students) (19.6% vs. 24.5%), and fewer reported other sources of income (19.0% vs. 12.6%).

**Table 1:** Description and comparisons of the sample.

Varaible	Categories	Prior impl	After impl	*p*
*N*		988	572	
Age [mean (SD)]		35.12 (13.50)	33.96 (12.22)	93
Gender [*n* (%)]	Female	683 (69.1)	395 (69.1)	1.000
	Male	305 (30.9)	177 (30.9)	
Have children [*n* (%)]	No	539 (54.6)	308 (53.8)	0.827
	Yes	449 (45.4)	264 (46.2)	
Marital Status [*n* (%)]	Single	399 (40.4)	219 (38.4)	0.171
	In relationship and living together	469 (47.5)	296 (51.8)	
	In relationship but living alone	120 (12.1)	56 (9.8)	
Highest level of Education [*n* (%)]	Primary school (0–9th grade)	142 (14.4)	65 (11.4)	<0.001***
	High school (10–12th grade)	189 (19.1)	140 (24.5)	
	Vocational education	106 (10.7)	68 (11.9)	
	Short further education (≤ 3 years)	113 (11.4)	68 (11.9)	
	Intermediate further education (4 or 5 years)	220 (22.3)	148 (25.9)	
	Long further education (≥ 5 years)	124 (12.6)	60 (10.5)	
	Other	94 (9.5)	22 (3.9)	
Source of income [*n* (%)]	Employed	333 (33.7)	234 (40.9)	<0.001***
	Social security	97 (9.8)	44 (7.7)	
	Sickness benefit/pay	67 (6.8)	40 (7.0)	
	Unemployment benefit	45 (4.6)	16 (2.8)	
	Stipendium	194 (19.6)	140 (24.5)	
	Pension	64 (6.5)	26 (4.5)	
	Other	188 (19.0)	72 (12.6)	

Prior impl, Sample prior to implementation of new screening tool; After impl, Sample after implementation of new screening tool.

* *p *< .05; **p < .01; ***p < .001.

### Consequences of implementing the new screening procedure

#### Primary hypothesis

**Figure 2:**
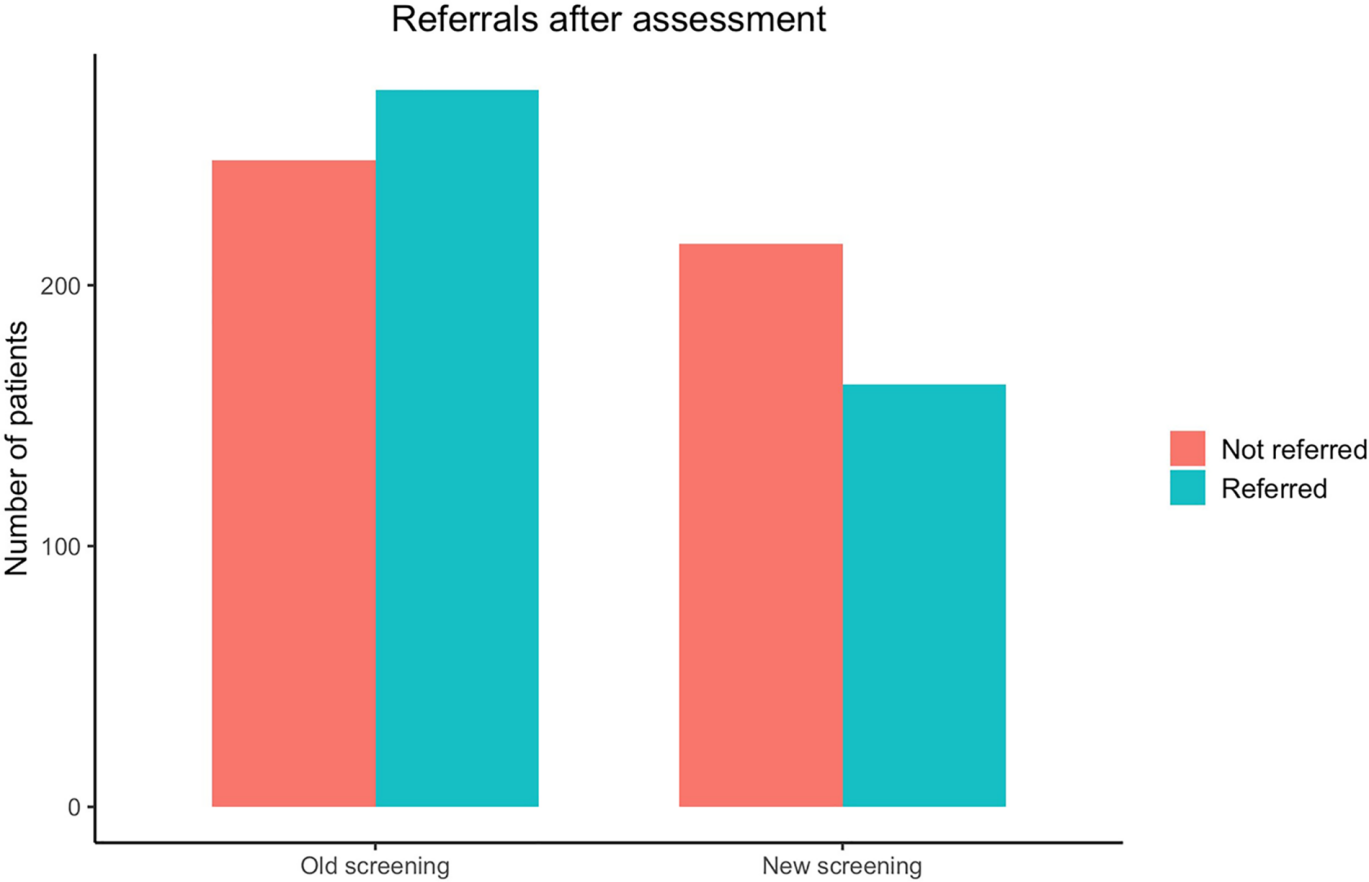
Number of patients referred to treatment after assessment.

As can be seen in Figure 2, contrary to Hypothesis 1, proportionately fewer applicants were referred to treatment following the assessment interview after the implementation of the new screening procedure. Before implementation, 52.58% were referred to treatment compared to only 42.86% after. When modeled with logistic regression, there was a significant difference between the two samples with an odds ratio of 0.67 (95% CI 0.51;0.87), meaning the odds of being referred after implementation of the new screening procedure is approximately a third lower. The proportions and the results of the logistic regression models can be seen in Table 2.

**Table 2:** Results of screening on initiation of iCBT treatment.

	Number of patients assessed				
	Old screening (*n* = 523)	New screening (*n* = 378)	Difference in %-points	Odds ratio (95% CI)	*p-*value
Assessed *and* referred to treatment	275 (52.58%)	162 (42.86%)	−9.72		
Assessed but *not* referred to treatment	248 (47.42%)	216 (57.14%)	9.72		
ANCOVA					
Unadjusted				0.68 (0.52;0.88)	0.004**
Adjusted				0.67 (0.51;0.87)	0.003**

*p < .05; **p < .01; ***p < .001.

#### Second hypothesis

**Figure 3:**
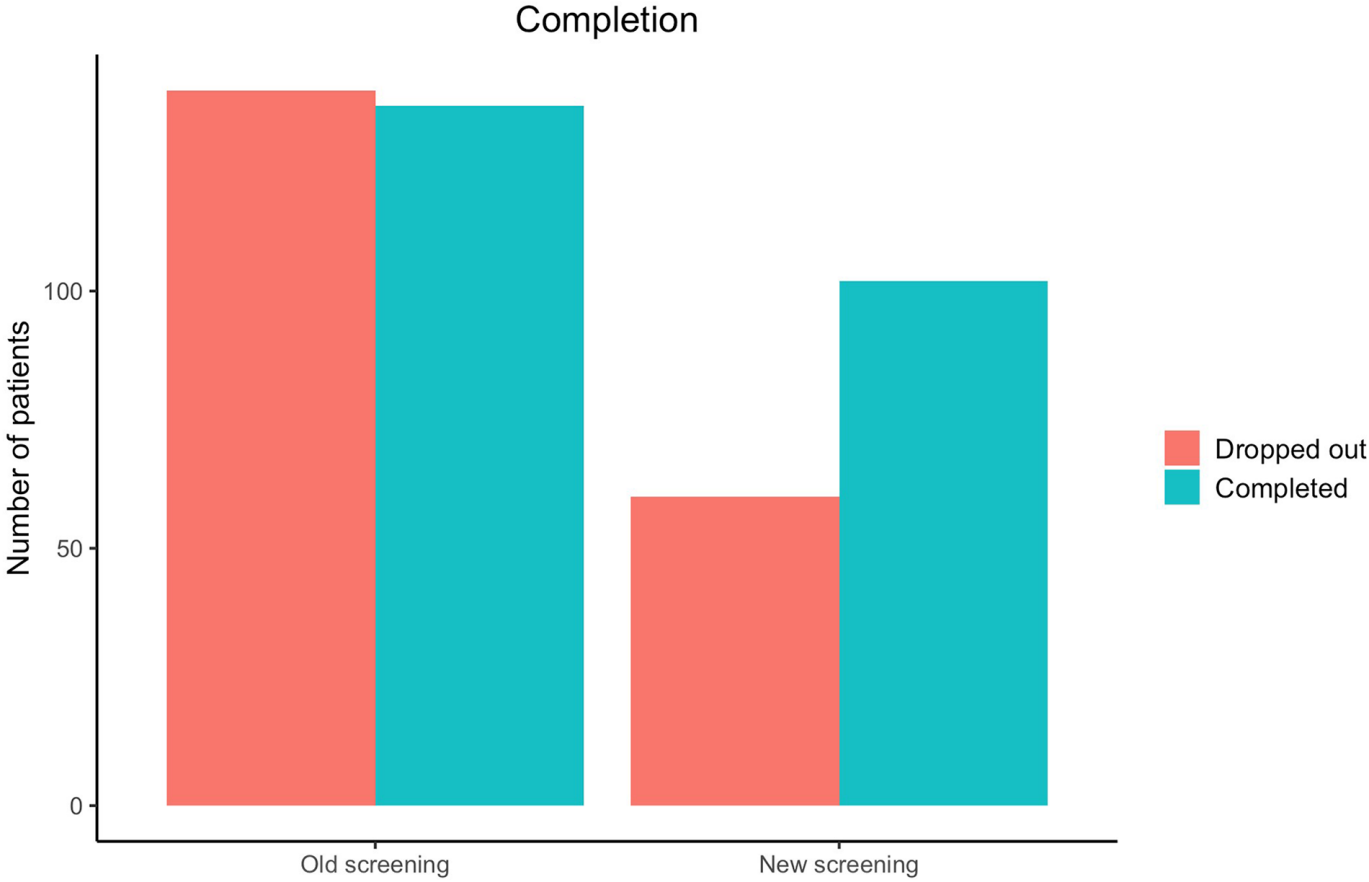
Number of patients completing treatment after being referred.

As can be seen in Figure 3, in line with Hypothesis 2, after implementation of the new screening procedure, a larger proportion of those referred to treatment also completed it (49.45% VS. 62.96%). When modeling these results with logistic regression analysis, the odds ratio of completing treatment after implementation of the new screening procedure is 1.79 (95% CI 1.20;2.70, *p *= 0.005), meaning the patients are approximately three-quarters more likely to complete the treatment. This difference is highly significant. The proportions and results of the logistic regression can be seen in Table 3.

**Table 3:** Results of screening on completion.

	Number of patients (n, %)				
	Referred to treatment, old screening (*n* = 275)	Referred to treatment, new screening (*n* = 162)	Difference in %-points	Odds ratio (95% CI)	*p-*value
Referred to treatment and *completed*	136 (49.45%)	102 (62.96%)	13.51		
Referred to treatment but *did not* complete	139 (50.55%)	60 (37.04%)	−13.51		
ANCOVA					
Unadjusted				1.74 (1.17;2.59)	0.006**
Adjusted				1.79 (1.20;2.70)	0.005**

*p < .05; **p < .01; ***p < .001.

#### Third hypothesis

**Figure 4:**
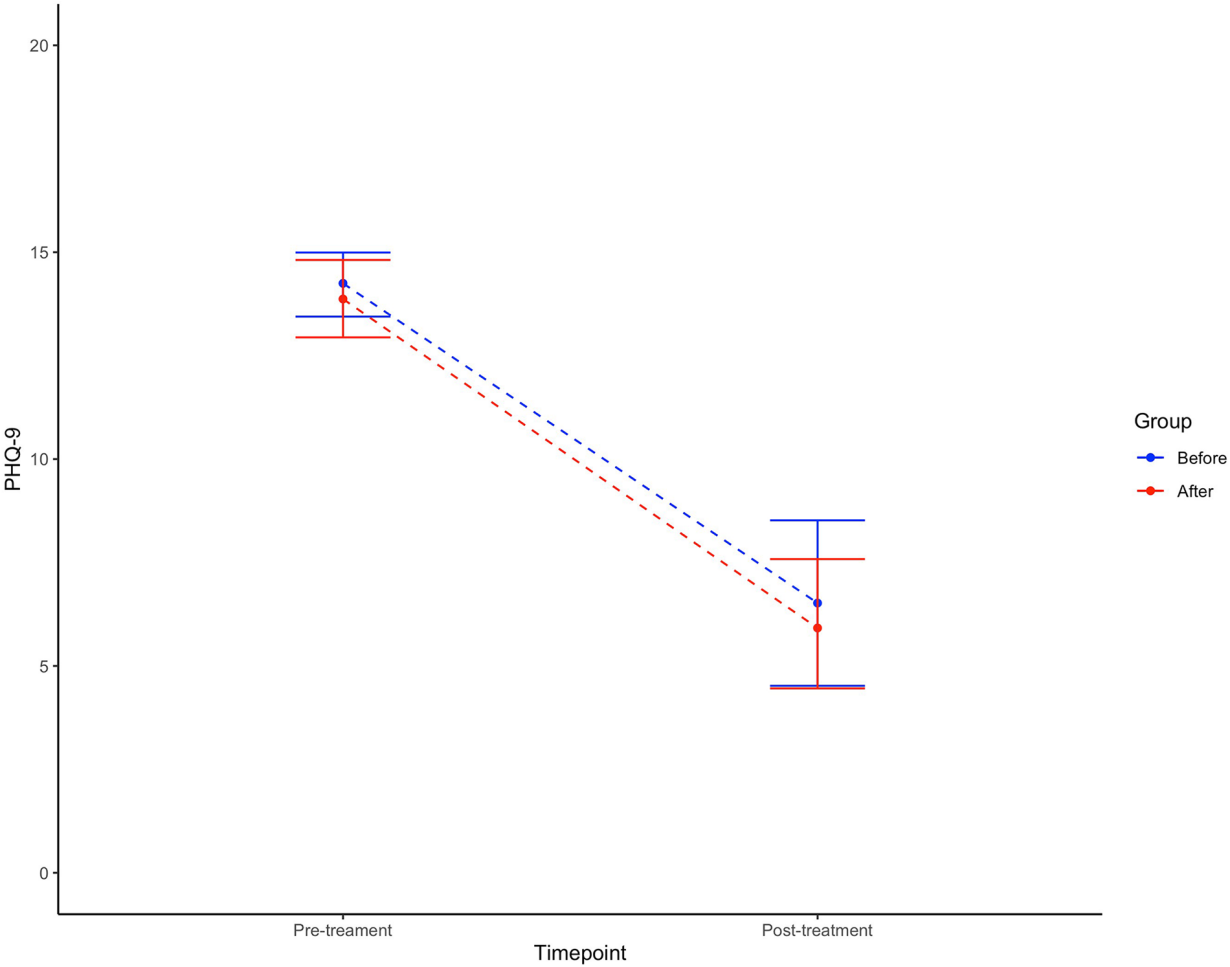
Change in levels of depressive symptoms during treatment.

Figure 4 shows the symptom reduction for depressed patients during treatment from pre-treatment to post-treatment for the two samples, respectively. Before the implementation the mean PHQ-9 score at baseline was 15.83 (SD 5.04) and 6.52 (SD 5.11) post-treatment, a significant within-group reduction (*t* = 8.4318, df = 32.635, *p < *0.001). A nearly identical development of symptoms was seen after implementation of the new screening procedure with a drop in mean PHQ-9 from 15.06 (SD 4.77) to 6.60 (SD 5.15), also significant (*t* = 7.2997, df = 37.758, *p *< 0.001). It is worth noting that the PHQ-9 cut-off for severe depression is at 15. However, contrary to Hypothesis 3, no difference was seen between the groups (0.01, 95% CI −2.73;2.75, *p *= 0.996). Table 4 describes the results.

**Table 4:** Results of screening on symptom reduction for depression.

	Symptom scores [mean (SD)]			
	Old screening (*n* = 149)	New screening (*n* = 87)	Difference of differences (95% CI)	*p-*value
Depression (*n* = 236)				
Baseline (PHQ-9)	15.83 (SD = 5.04, *n* = 144)	15.06 (SD = 4.77, *n* = 80)		
Post-treatment (PHQ-9)	6.52 (SD = 5.11, *n* = 23)	6.60 (5.15, *n* = 21)		
ANCOVA				
Unadjusted			0.56 (−2.83;3.95)	0.741
Adjusted			0.01 (−2.73;2.75)	0.996

*p < .05; **p < .01; ***p < .001.

The GAD-7 was not included in the clinics data collection until 5 November 2019, which is within the study period before implementation of the new screening procedure. As a result, too few are included with a GAD-7 score to perform the analyses. This too was the case for each of the diagnosis specific questionnaires, which splits the sample into too small sub-groups.

## Discussion

This study aimed to investigate, whether by using a self-guided novel implementation toolkit (the ItFits-toolkit) a local group of implementers managed to identify and achieve an implementation goal set in the local context i.e., tailored implementation. The Danish national iCBT clinic Internetpsykiatrien wished to develop a novel online screening tool with the aim of including a more relevant sample for assessment and treatment in the clinic. This was investigated in the present study by examining three hypotheses: (1) a larger proportion of assessed patients were referred to iCBT treatment, (2) as a consequence of including a more relevant sample, the completion rate was increased, and finally (3) for the same reason, effectiveness was increased.

### Intake before and after

Contrary to our first hypothesis, fewer video-assessments resulted in referral to treatment after implementation of the new screening tool. The odds of being referred to treatment after implementation of the new screening procedure was approximately a third lower than before. A possible explanation could be that the implementation process increased awareness and mutual understanding of the inclusion and exclusion criteria among the clinical staff. This could result in less individual path dependency and more rational assessments based on standard criteria. It could be said that clearer criteria were communicated via the implementation process, streamlining intake, which could explain the decrease in intake. This would indicate that prior to implementation, assessors showed more leniency regarding the criteria, and applicants who did not match the inclusion criteria could still be included. This is unfortunately beyond the scope of the present study to examine. Future studies on similar implementation processes should gather more information on the patients invited to assessments to get a clearer picture of the whole patient demographic assessed. For the present study we do not know whether the implementation process affected (1) the demographic of the patients that were referred to treatment, (2) the demographic of the patients that were excluded, or (3) both those referred to treatment and those excluded during the video assessment.

Additionally, the lower number of self-referrals might be explained by the added information at the start of the online screening tool. Many people may not have been fully aware of what iCBT is. When properly informed about the treatment at the start of the screening process, some people may stop filling out the questionnaire, if they realize they prefer a different kind of treatment. An interesting number that may corroborate this hypothesis is the large number of canceled video assessments prior to implementation. Prior to implementation 246 (30.8%) out of 799 approved assessments were canceled by the applicants. After implementation, this figure dropped to 39 (8.3%) out of 471. This could be an indication that prior to implementation, more applicants did not realize what iCBT treatment was until after self-referral and therefore canceled their appointment. This may also explain the reduced number of people with lower educational background. The high degree of reading and homework required to benefit from a written online treatment may discourage people who are not bookish from applying to the program.

### Drop-out rates before and after

The second hypothesis was supported by the results. After implementation, a larger proportion of those referred to treatment completed the treatment compared with the period prior to implementation.

The higher rate of completion post-implementation lends support to the hypothesis that more ineligible patients were offered treatment pre-implementation. Eligible patients would likely find the treatment content more relevant, and be more likely to complete the treatment.

The proportion of completed treatments prior to implementation of the new online screening tool was relatively low. After implementation the number of completed treatments improved, although 62.96% is still slightly on the low side compared with the field as a whole. Melville, Casey & Kavanagh (2010) reported an average adherence rate of 69% in a review of internet-based treatments for psychological disorders (36), and Andrews and colleagues (2018) reported a median adherence rate of 66% in a review of internet-based treatment for anxiety and depression (37). Studies vary in their definitions of adherence and drop-out, which could influence these figures (36). However, it is clear that adherence could still be improved significantly.

### Depression scores pre- and post-implementation

Finally, we hypothesized that the new screening tool resulted in larger symptom reductions. The results showed no difference between the treatment effects, demonstrating that treatment is equally effective in the pre- and post-implementation sample.

It seems that the screening tool did increase inclusion of the eligible target demographic, but this only affected completion. The patients that completed treatment prior to implementation were most likely already among the eligible target demographic and therefore showed symptom reductions comparable to the patients that completed treatment after implementation. Therefore, even though average treatment effects were comparable, it is worth noting, that a larger proportion of patients benefited from the full treatment effect after implementation of the new screening tool.

When interpreting the results on treatment effectiveness, it should also be considered that the treatment effect could only be examined among completers of the treatment programs that replied to the post-treatment questionnaire. Therefore, we do not know if the new screening tool resulted in changes in treatment effectiveness among non-completers. Further, we did not have enough data to run an analysis on the anxiety patient sample. Thus, these results only apply to patients offered treatment for depression.

### Additional observations

When looking at the numbers for how many applicants were deemed eligible for a video assessment, there is a substantial drop from the pre- to post-implementation. This is potentially worrying because it indicates that the screening has possibly become too hard, thereby screening out people who may have benefitted from the program. However, the higher completion rate still indicates that the post-screening is more apt at including the target demographic. And this feeds into the ethical debate regarding the balance between cost and benefit of therapy. Because even though fewer are included, and some who may have benefitted are excluded, the benefits here outweigh the negatives. If a patient is included in a program, they do not benefit from, they may be discouraged from further help-seeking. Further, if too many resources are used in vain, they cannot be used on other patients. Lastly, the therapists may not be able to help the patient in a meaningful way, increasing the risk of frustration and negative experiences for the clinical staff.

### Practical implications

The study underlines some points to be aware of when optimizing screening procedures in the iCBT clinic.

***Aligning expectations:*** Communicating clearly about the purpose and style of iCBT even before the potential patient has had any contact with staff, can ensure that neither clinicians nor patients waste their time on a treatment method, which may not be suitable for that specific patient.
***Balancing inclusion and exclusion:*** Finding the balance between inclusion and exclusion criteria that does not exclude too many potential benefiters, but does not waste therapist resources either.
***Reaching consensus between therapists:*** Differences in therapists’ consensus on treatment may be moderating or confounding effects during new interventions.

## Strength and limitations

Some limitations for this study should be mentioned. First, this study was heavily reliant on the registration praxis of the clinicians working at the clinic at the time. The consistency of this praxis could be questioned, which was also the case for the 70 applications that were excluded from analyses. However, even though inconsistencies in registration are bound to occur, the general picture will most likely be correct for a sample of this size. Second, there was insufficient data to run an analysis on symptom reduction for patients offered treatment for anxiety. Therefore, we cannot say whether improved intake affected symptom reduction for this group. This is unfortunate, especially since the improved completion rates were more pronounced for anxiety compared with depression (16.7%-points vs. 10.7%-points more completed treatments). However, if our assumptions regarding treatment completers prior to implementation being those from the target demographic are true, we would not expect this analysis to show anything different from the analysis on depression treatment effect. We also do not know how the new screening tool changed the user experience of the screening, which may have influenced intake. Lastly, we were only able to analyze treatment effects for completers. It would have been interesting to see, if the treatment effects were different among non-completers pre- and post-implementation, as this would have provided further information on whether the screening tool successfully included the target demographic, even among non-completers.

## Conclusion

The revised screening tool investigated in this study was meant to improve the eligibility of self-referrals for treatment at Internetpsykiatrien. Whether this was achieved is somewhat unclear. The number of self-referrals dropped in the 6 months after implementation compared to the 6 months prior to implementation. Likewise, the proportion of assessment interviews that resulted in a referral to treatment was reduced. However, the proportion of patients referred to treatment that completed the full treatment increased. No difference was found for treatment effects. We interpret these results as an indication that the screening tool did result in more relevant self-referrals and assessment interviews. Thus the toolkit was an effective means of aiding a tailored local implementation effort. However, the effect on the assessment interviews may be masked by a simultaneous increase in awareness of and stringency with the inclusion and exclusion criteria for treatment among the clinicians.

## Data Availability

The original contributions presented in the study are included in the article, further inquiries can be directed to the corresponding author.
